# An updated audit of the patient selection process for pain management programmes in a speciality care service before and during the COVID-19 pandemic

**DOI:** 10.1177/20494637221147200

**Published:** 2022-12-23

**Authors:** Valentina Buscemi, Joe Chicken, Tim Mahy, Lucie Knight, Whitney Scott

**Affiliations:** 1 INPUT Pain Management Unit, 8945 Guy’s and St Thomas’ NHS Foundation Trust, London, UK; 2 Health Psychology Section, Institute of Psychiatry, Psychology, and Neuroscience, 4616 King’s College London, London, UK

**Keywords:** Chronic pain, Pain management, Acceptance and commitment therapy, Patient selection, audit

## Abstract

**Background:**

The provision of pain management programmes (PMPs) changed substantially in response to the COVID-19 pandemic with virtual delivery implemented in many services. Little is known about patient selection processes for virtual PMPs and how this might differ from in-person programmes. The aim of this audit was to document the patient selection process for PMPs at a speciality pain service prior to and during the pandemic.

**Methods:**

This retrospective audit used data from consecutive patients attending a multidisciplinary assessment to determine the suitability of a PMP. Anonymized data were extracted from assessment letters and hospital records in the months prior to the pandemic (n =168) and during the start of the pandemic once the service began delivering virtual PMPs (n =171).

**Results:**

For the standard pain management pathway, most patients were offered a PMP option within the service before and during the pandemic, although a greater proportion of patients were offered treatment during the pandemic. For the neuromodulation pathway, most patients were offered a pre-neuromodulation PMP option, and this was similar before and during the pandemic. Psychosocial complexities and unwillingness to engage in a pain management approach that does not principally focus on pain reduction were the most common reasons that patients were not offered a programme.

**Discussion:**

This audit points to a pattern of more inclusive assessment outcomes within our service over time and particularly during the pandemic. Offering a range of in-person and virtual PMPs can meet a wider range of patient need. Research is needed to understand how to best assess and match patients with the breadth of treatment delivery formats now available.

## Introduction

Pain Management Programmes (PMPs) have been offered for people with persistent pain for many years and numerous studies support their effectiveness.^1-7^ The aim of PMPs is not pain reduction. Rather, multidisciplinary programmes focus on cognitive-behavioural self-management skills enabling participants to engage in valued activities in the face of pain and other challenges.^1^ PMPs have been offered in different formats to meet a range of patients’ needs, from intensive residential to less intensive outpatient formats.^8,9^ PMPs have also been offered for patients deemed medically suitable for neuromodulation.^10^ PMPs prior to neuromodulation provide participants with information about this intervention, so they can make an informed decision about their treatment together with the multidisciplinary team. However, the main aim is to introduce self-management skills to give patients the tools to respond to their pain differently, regardless of the outcome of neuromodulation.^10^ Most PMPs have traditionally been group-based and offered in person.^11^

PMPs are not deemed suitable for all people with persistent pain^1^ and better understanding of patterns of exclusion can help to develop more inclusive treatment options. To this end, in 2014 our tertiary pain service conducted an audit of outcomes for 200 patients attending multidisciplinary assessments to judge the suitability of a PMP.^12^ Just over half (53%) of the patients assessed were offered treatment, most frequently an intensive residential PMP. Some were offered a shorter outpatient PMP or individual pain management psychology sessions. For those patients who were not offered any of the treatments available within the service, alternative recommendations were frequently made, such as referral for psychological therapy for trauma or other complex psychological issues that were judged as likely to interfere with a person’s capacity to safely engage in a PMP. A sizeable group of patients were not offered a PMP because they were still seeking medical treatments to reduce pain. Some patients were excluded because they did not meet the minimum physical criteria to attend a residential PMP, which typically requires patients to be independently self-caring. On the other end of the spectrum, there was a group of patients who were functioning relatively well and did not need a PMP.^12^ This audit highlighted potential gaps in service provision for a number of patients. Notably, this audit did not examine assessment outcomes for patients that are potentially suitable for a PMP to prepare for neuromodulation. Therefore, less is known about assessment outcomes for patients on this pathway.

With the onset of the COVID-19 pandemic in early 2020, practically all pain services in the UK had to stop offering in-person PMPs because of the public health situation. Considerable effort went into rapidly developing alternatives and soon virtual options for assessment and treatment delivery were trialled.^13,14^ Within this context, our service redeveloped a number of previous in-person PMPs for virtual delivery, which included high and low intensity virtual PMPs and a virtual neuromodulation PMP, among other options. These virtual programmes were designed to meet the needs of patients previously recommended our in-person residential, outpatient and neuromodulation PMPs, with the more intensive PMPs designed to support people with more severe or complex pain-related disability and/or distress than the lower intensity PMP. Prior to the pandemic, our service also offered an online PMP which consisted of website-based pre-recorded audio and video content that patients access asynchronously with minimal therapist support,^15^ consistent with a growing body of evidence supporting Internet-delivered cognitive-behavioural PMPs.^16,17^ While this online programme can increase access given its flexibility, particularly for patients who are working or have caring responsibilities, it may not be suitable for patients needing more intensive support, such as those presenting with greater severity of pain-related disability or distress. To provide more intensive support for patients presenting with more severe pain-related disability and/or distress, such as those who would have been offered a residential or outpatient programme before the pandemic, our service developed virtual PMPs delivered synchronously with clinician facilitation.

With this changing landscape of pain management services, it is crucial to consider how virtual options impact on accessibility and patient selection processes. Of course, virtual programmes have advantages.^18,19^ Many people with chronic pain struggle using public transport, driving, or walking long distances. Attending from home, therefore, makes programmes available to participants who might previously have been excluded from in-person programmes. Virtual options require less space to run and come with efficiencies that are attractive to commissioners. Staff can run virtual programmes from home or on site, thus increasing flexibility.

One obvious drawback of virtual options is their reliance on technology, which not everyone has access to.^18^ For example, people of lower socio-economic status might not have a computer or Internet access and not everyone has sufficient digital literacy to manage a virtual PMP, which may exclude the very people in most need of support.^20^ Lack of stable Internet connections for both patients and clinicians can also pose a problem.^19^ In-person programmes offer opportunities to observe participants directly, including their movements, functional abilities, reactions to interventions and body language. This is limited when working virtually, which means clinicians might find it more challenging to connect with patients in a deep and meaningful way.^18^ Although functionalities such as ‘breakout rooms’ can enable treatment participants to share their thoughts and feelings, this may disrupt the natural flow of communication and engagement that happens during in-person treatments. Additionally, participants can experience screen fatigue,^13^ limiting the length and number of virtual sessions.

The rapid changes to service delivery that occurred because of the pandemic provided a unique opportunity to investigate how the move from in-person to virtual PMPs affected patient selection processes. The aim of the current audit was to repeat the previous audit on patient selection for PMPs by Knight et al.^12^ in the context of programme adaptations that were made during the pandemic. The current audit also builds on the previous one by examining assessment outcomes and exclusion reasons for patients on our neuromodulation pathway before and during the pandemic. Across pathways and time periods, clinician recommendations and reasons for exclusion were identified using information provided in assessment reports. Patient demographics were examined in relation to whether a person was offered treatment or not. Data from this audit will help to inform service provision and options for inclusively delivering PMPs moving forward.

## Methods

This project was an audit based on the Health Research Authority’s decision tool, as it was designed to measure current practices within the service against a previous standard and was not designed to be generalizable. Therefore, research ethics approval was not required. This retrospective audit was registered on the Trust’s audit database (no. 11336) prior to data collection. The audit used data from consecutive patients attending a specialist pain management service in London, UK for a multidisciplinary assessment to determine the suitability of a PMP. Data were extracted from assessment letters and hospital records prior to the COVID-19 pandemic (December 2019 to early March 2020; *n* = 168) and during the pandemic once the service began delivering virtual PMPs (June–August 2020; *n* = 171). Extracted data were anonymized and stored in a secured database that was only accessed by members of the audit team who were all working clinically in the service. As this was a retrospective audit of routine clinical practice using anonymized data, informed consent was not obtained, as approved by the Trust audit register.

Prior to the pandemic, patients were considered for a three-week residential PMP, a five-session outpatient PMP, or an online self-directed PMP that was completed via a website with asynchronous therapist support. Short-term individual psychology/physiotherapy treatment was offered for people for whom group-based treatment was not suitable, including due to the need for an interpreter or significant interpersonal challenges, such as severe social anxiety, that would impact on their ability to engage with a group-based format. All of these treatment options were based on Acceptance and Commitment Therapy (ACT) and aimed to foster psychological flexibility to help people engage in meaningful life activities in the presence of pain.^21^ Patients deemed medically suitable for spinal cord stimulation (SCS) were considered for a two-week residential neuromodulation PMP or a one day ‘Technology Day’; both of these programmes prepare people to make a fully informed decision about SCS, while the two-week programme also focuses on the development of ACT-based pain management strategies. The choice between the two neuromodulation programmes is predominantly based on clinicians’ judgement of the level of pain-related disability/distress, although patients’ preference and considerations including childcare and work commitments, also influence the recommendation. Patients could also be recommended for ‘case management’ to provide extra support to help them prepare to optimally engage with one of these PMPs as needed (e.g., medication reduction, viewing the accommodation before a residential programme). Further details about the nature of and outcomes associated with these programmes have previously been published.^9,10,15^

During the pandemic, patients were also considered for synchronously delivered virtual high and low intensity PMPs, virtual individual psychology/physiotherapy sessions, or a virtual two-week neuromodulation programme or Technology Day (if medically suitable for SCS). The synchronous virtual programmes were delivered by the BlueJeans/Attend Anywhere platforms approved by the NHS Trust where the audit was conducted. Table 1 provides a summary of the delivery format of the different treatment options for patients before and during the pandemic.

**Table 1.** Summary of PMP delivery formats offered before and during the pandemic.

Type of pain management programme	Treatment days and duration	Clinicians involved	When considered at assessment
Residential (in person)	15 days over 3 weeks	Psychologist Physiotherapist Occupational therapist Nurse	Pre-pandemic During pandemic*
Outpatient (in person)	5 days over 3 weeks	Psychologist Physiotherapist	Pre-pandemic During pandemic*
Individual psychology/physiotherapy (in person)	Up to 8 one-hour sessions	Psychologist and/or Physiotherapist	Pre-pandemic During pandemic*
Asynchronous online PMP with minimal therapist support	2 calls and 8 self-paced sessions over 5 weeks	Psychologist or Physiotherapist	Pre-pandemic During pandemic
Residential neuromodulation (in person)	8 days over two weeks	Psychologist Physiotherapist Occupational Therapist Nurse	Pre-pandemic During pandemic*
Neuromodulation ‘Technology Day’ (in person)	1 day	Psychologist Physiotherapist Nurse	Pre-pandemic During pandemic*
High intensity virtual (synchronous delivery)	12 days over 3 weeks	Psychologist Physiotherapist Occupational Therapist Nurse	During pandemic
Low intensity virtual (synchronous delivery)	5 days over 3 weeks	Psychologist Physiotherapist	During pandemic
Individual virtual psychology/physiotherapy (synchronous delivery)	Up to 8 one-hour sessions	Psychologist and/or Physiotherapist	During pandemic
High intensity virtual neuromodulation (synchronous delivery)	8 days over 2 weeks	Psychologist Physiotherapist Occupational therapist Nurse	During pandemic
Virtual neuromodulation ‘Technology Day’ (synchronous delivery)	1 day	Psychologist Physiotherapist Nurse	During pandemic

^*^The option for in-person programmes was still considered for some people during the pandemic if they were unable to access a virtual programme. In such cases, patients were informed of the uncertainty of the timeframe for when in-person treatment would resume and they were placed on the waiting list for this for when on site delivery was allowed according to local approvals.

The multidisciplinary assessments were conducted by a psychologist and physiotherapist to judge the suitability of an ACT-based pain management programme and, where not suitable, to make recommendations about support needed from other services. Across all programmes (in-person and virtual), the general inclusion criteria were adults (18 years or older) with pain for at least three months that significantly impacted on daily function and/or mood. The general exclusion criteria across treatment formats included significant ongoing medical investigations or procedures (with the exception of neuromodulation) and psychosocial complexities that would impact safe and effective engagement in a PMP (e.g. active suicidality, unstable housing). Additionally, individuals with substance misuse or addiction, including to prescription opioids, that were the overriding focus of the presenting problem were excluded from the programme.

For residential programmes, patients had to be able to manage their own self-care and the physical requirements while staying on site. For virtual programmes, patients were required to have Wi-Fi access and a private space to use while completing the programme. The service was able to loan a tablet for the purpose of completing the programme for those with Wi-Fi access but no suitable device. For security purposes, the loaned tablets were configured such that participants could only access the ‘BlueJeans’ video platform which was approved for use for the virtual PMPs by the Trust and pre-downloaded onto the devices. A dedicated courier from the Trust delivered the tablets to ensure participants received them in advance of the programme.

Decisions about treatment intensity and format (e.g. group-based versus individual) were made based on patients’ needs. The more intensive programmes (i.e. residential or high intensity virtual) are offered for patients with greater severity/complexity of pain-related disability and/or distress, while the lower intensity treatments (i.e. in-person outpatient or low intensity virtual) are typically offered for patients with less severe disability and/or distress. The multidisciplinary assessment consists of a semi-structured interview and the clinicians make a judgement about the suitability of a PMP considering the complexity of information from this interview. Standardized pain and psychosocial questionnaires are not administered as part of this assessment and therefore are not captured in the assessment letters.

### Data collection procedure

Assessment letters were reviewed for each patient that had an assessment during the dates identified above. A standardized data extraction form was developed based on the previous audit^12^ and refined to reflect the changes in service provision that occurred since that audit. Main pain location and pain duration were extracted from the assessment letter where reported. Patient age, gender and ethnicity were extracted from the hospital registration system where this information was recorded. Assessment time period (pre-COVID and during COVID) and pathway (standard PMP or neuromodulation pathway) were recorded on the data extraction form. Assessment outcomes were recorded using the following categories:

1) Three-week residential PMP
2) High intensity virtual PMP
3) Outpatient PMP
4) Low intensity virtual PMP
5) Online PMP (self-directed with therapist support)
6) Individual psychology in-person
7) Individual psychology virtual
8) Individual physiotherapy in-person
9) Individual physiotherapy virtual
10) Joint individual psychology and physiotherapy in-person
11) Joint individual psychology and physiotherapy virtual
12) Two-week residential neuromodulation PMP
13) Two-week virtual neuromodulation PMP
14) Neuromodulation Technology Day in-person
15) Neuromodulation Technology Day virtual
16) Referral to internal pain consultant
17) Discharge
18) Other (specify)

The data extraction form also captured whether patients were recommended for ‘case management’ through the service’s nursing and/or occupational therapy teams to prepare them to attend one of the recommended programmes. For patients that were discharged, reasons for discharge were recorded using the following categories which reflect key inclusion/exclusion criteria for the programmes, as summarized earlier:

1) Does not meet minimum physical criteria
2) Psychosocial complexities limiting ability to engage safely and effectively
3) Addiction/substance misuse
4) Not ready to engage in self-management/seeking further interventions
5) Pain is not significantly impacting on functioning/quality of life
6) Unable to participate in English
7) Other (specify)

The reasons for discharge were not mutually exclusive and multiple reasons could be recorded on the data extraction form if indicated in the assessment letter. If ‘psychosocial complexities’ was selected as a discharge reason, the specific details of this were also recorded (e.g. active suicidality, severe/untreated post-traumatic stress disorder, unstable housing). Finally, for discharged patients, recommendations made for onward support in the assessment letter were extracted.

The data extraction was split between four individuals working clinically in the service who had experience with the assessment process and understanding of the PMPs offered. Prior to commencing the main data extraction, the four data extractors piloted the extraction form with the same twenty patients for training purposes. The pilot data extraction was compared across the four extractors and discrepancies were discussed to ensure clarity and consistency of coding. Refinements to the data extraction form were made as necessary to further ensure clarity and consistency. For example, there was initial inconsistency in the application of the coding for main pain location with respect to widespread pain. Following discussion amongst the data extraction team, it was agreed to use the code of widespread pain for assessment letters using the terms ‘widespread pain’ or ‘fibromyalgia’, and where the letters described that patients reported three or more pain locations.

### Data analysis

Descriptive statistics were computed for demographic variables by treatment pathway (standard PMP or neuromodulation) and time period (pre-COVID or during COVID). Frequencies were computed for each assessment outcome according to treatment pathway and time period. Where the assessment outcome was to discharge the patient, frequencies were computed for the discharge reasons, including frequencies of specific psychosocial complexities documented as a reason for discharge. Finally, frequencies were computed for the recommendations made for onward support for discharged patients.

Chi-square tests were conducted to compare the proportion of patients discharged before and during the pandemic for the standard PMP and neuromodulation pathways. Chi-square tests were also used to compare whether discharge rates varied by patient ethnicity, coded as ‘white’ or ‘from an ethnically minoritized background’. Mann–Whitney U tests were computed to compare whether people who were and were not discharged differed in terms of age and pain duration, which were not normally distributed. Given the relatively small number of patients that were discharged, patient discharge status was collapsed across treatment pathway and time period for these analyses.

## Results

Demographic characteristics of patients included in the audit by pathway and time period are presented in Table 2. Patients were predominantly women (67–80%), except for patients attending assessment for the neuromodulation pathway before the pandemic, which were predominantly men (56%). Patients’ median age ranged from 48 to 51.5 years (18–84). Patients had pain of longstanding duration, with a minimum median duration of 7 years (1–51) across time period and pathways. There was a high proportion of missing data for ethnicity (49–74% missing overall). Among those who did have ethnicity data recorded, patients were predominantly white, ranging from 63% in the standard PMP pathway during the pandemic to 100% white for the neuromodulation pathway pre-pandemic. Widespread pain was the most common for patients assessed as part of the standard PMP pathway, while low back and limb pain was the most common for patients on the neuromodulation pathway.

**Table 2.** Demographics and pain characteristics by pathway and period.

Variable	Pre-COVID: Standard MDT pathway (*n* = 100) Frequency(%)*	During COVID: Standard MDT Pathway (*n* = 128) Frequency (%)	Pre-COVID: NM pathway (*n* = 68) Frequency (%)*	During COVID: NM pathway (*n* = 43) Frequency (%)
Gender				
Women	80 (80.0)	97 (75.8)	30 (44.1)	29 (67.4)
Men	19 (19.0)	31 (24.2)	39 (55.9)	14 (32.6)
Missing	1 (1.0 overall)	0 (0.0)	0 (0.0)	0 (0.0)
Ethnicity				
Asian/Asian British: Indian	0 (0.0)	3 (4.6)	0 (0)	0 (0)
Asian/Asian British AOB	1 (2.0)	0 (0.0)	1 (5.6)	0 (0)
Black/Black British: African	0 (0.00)	5 (7.7)	0 (0)	1 (6.7)
Black/Black British: Caribbean	5 (10.0)	5 (7.7)	0 (0)	1 (6.7)
Black/Black British AOB	10 (20.0)	8 (12.3)	0 (0)	1 (6.7)
Mixed: White/Black Caribbean	0 (0.0)	1 (1.5)	0 (0)	0 (0.0)
Mixed: AOB	0 (0%)	1 (1.5)	0 (0)	1 (6.7)
White British	21 (41.0)	34 (52.3)	17 (94.4)	9 (60.0)
White AOB	13 (25.0)	7 (10.8)	0 (0)	2 (13.3)
AOB not already described	1 (2.0)	1 (1.5)	0 (0)	0 (0.0)
Missing	49 (49.0 overall)†	63 (49.2 overall)†	50 (73.5 overall)†	28 (65.1 overall)†
Ethnicity collapsed				
Ethnically minoritized	17 (33.3)	24 (36.9)	1 (5.6)	4 (26.7)
White	34 (66.7)	41 (63.1)	17 (94.4)	11 (73.3)
Missing	49 (49.0 overall)†	63 (49.2 overall)†	52 (76.4 overall)†	28 (65.1 overall)†
Age (years)	48 (22–84)	48 (18–74)	51.5 (24–76)	50 (21–74)
Main pain location				
Widespread	61 (61.0)	92 (71.9)	0 (0.0)	7 (16.3)
Lower back	16 (16.0)	13 (10.2)	32 (47.1)	11 (25.6)
Spinal (other than lower back)	7 (7.0)	8 (6.3)	2 (2.9)	4 (9.3)
Neck	3 (3.0)	1 (0.8)	0 (0.0)	3 (7.0)
Upper limbs	3 (3.0)	1 (0.8)	8 (11.7)	5 (11.6)
Lower limbs	4 (4.0)	8 (6.3)	19 (27.9)	10 (23.3)
Chest	1 (1.0)	0 (0.0)	0 (0.0)	1 (2.3)
Abdomen	4 (4.0)	0 (0.0)	3 (4.4)	0 (0.0)
Genitals/pelvic	0 (0.0)	3 (2.3)	1 (1.5)	1 (2.3)
Head	0 (0.0)	1 (0.8)	3 (4.4)	1 (2.3)
Missing	1 (1.0 overall)	1 (0.8 overall)	0 (0.0 overall)	0 (0.0 overall)
Pain duration (years)	9 (1–51)	10 (2–39)	10 (2-30)	7 (1-35)

*Note:* AOB, Any other background; PMP, pain management programme; NM, neuromodulation pathway.

* Ethnicity had substantial missing data. Therefore, the denominator used to compute the percentage was based on the number of patients for whom this data was available; the percentage for all other variables used the total number of participants for the pathway/time period as the denominator.

† Percentage missing overall computed from the total number of patients assessed.

Assessment outcomes by treatment pathway and time period are shown in Table 3. For the standard PMP pathway, the most common programme recommended prior to the pandemic was the residential PMP (44%), while the high intensity virtual PMP was most commonly recommended during the pandemic (41%). Before the pandemic, similar proportions of patients were offered the two-week residential neuromodulation PMP (41%) and the ‘Technology Day’ (44%), while the virtual two-week neuromodulation and Technology Day programmes were recommended to 30% and 26% of patients on the neuromodulation pathway during the pandemic. During the pandemic, 20% and 26% of patients were still offered in-person programmes on the standard and neuromodulation PMP pathways, respectively.

**Table 3.** Assessment outcomes by pathway and period.

	Pre-COVID: Standard MDT pathway (*n* = 100) Frequency (%)	During COVID: Standard MDT pathway (*n* = 128) Frequency (%)	Pre-COVID: NM pathway (*n* = 68) Frequency (%)	During COVID: NM pathway (*n* = 43) Frequency (%)
**Assessment Outcome:**				
Discharge	31 (31.0)	21 (16.4)	3 (4.4)	2 (4.7)
Referral to internal consultant	0 (0.0)	2 (1.6)	2 (2.9)	2 (4.7)
**In-person treatments:**				
3-week residential PMP	44 (44.0)	16 (12.5)	N/A	N/A
Outpatient PMP	19 (19.0)	6 (4.7)	N/A	N/A
2-week residential PMP (NM)	N/A	N/A	28 (41.2)	6 (14.0)
Technology Day (NM)	N/A	N/A	30 (44.1)	5 (11.6)
Individual psychology (internal)	1 (1.0)	1 (0.8)	0 (0.0)	0 (0.0)
Individual psychology + physiotherapy (internal)	0 (0.0)	3 (2.3)	0 (0.0)	0 (0.0)
**Virtual treatments:**				
3-week high intensity PMP (synchronous)	N/A	52 (40.6)	N/A	N/A
3-week low intensity PMP (synchronous)	N/A	8 (6.3)	N/A	N/A
2-week high intensity PMP (NM; synchronous)	N/A	N/A	N/A	13 (30.2)
Technology Day (NM; synchronous)	N/A	N/A	N/A	11 (25.6)
Online PMP (low intensity, asynchronous)	2 (2.0)	1 (0.8)	0 (0.0)	0 (0.0)
Individual psychology (internal)	1 (1.0)	2 (1.6)	0 (0.0)	0 (0.0)
Individual psychology + physiotherapy (internal)	N/A	6 (4.7)	N/A	0 (0.0)
**Other Assessment Outcome:**				
Changed NM to MDT pathway (or vice versa)	0 (0.0)	0 (0.0)	1 (1.5)	1 (2.3)
Virtual high intensity PMP + future residential	0 (0.0)	1 (0.8)	N/A	N/A
2-week residential PMP (NM) + online PMP	N/A	N/A	1 (1.5)	0 (0.0)
Virtual Technology Day (NM) + online PMP	N/A	N/A	N/A	2 (4.7)
Discuss after mental health review^a^	1 (1.0)	5 (3.9)	0 (0.0)	0 (0.0)
Discuss after review of social circumstances	1 (1.0)	0 (0.0)	0 (0.0)	0 (0.0)
Discuss after further medical review	0 (0.0)	1 (0.8)	0 (0.0)	1 (2.3)
Straight to NM research trial	N/A	N/A	1 (1.5)	0 (0.0)
Extended assessment/time to think	0 (0.0)	2 (1.6)	2 (2.9)	(0.0)
3-week intensive in-person outpatient PMP	0 (0.0)	1 (0.8)	N/A	N/A
**Case Management** ^b^				
Occupational therapy	8 (8.0)	2 (1.6)	4 (5.9)	2 (4.7)
Nurse medication review	0 (0.0)	3 (2.3)	6 (8.8)	3 (7.0)
Occupational therapy + nurse medication review	0 (0.0)	1 (0.8)	0 (0.0)	0 (0.0)

*Note:* PMP, pain management programme; NM, neuromodulation pathway.

^a^For example, after completion of external psychological treatment.

^b^Case management recommended in conjunction with other treatment options.

The proportion of patients who were discharged from the standard pain management pathway before COVID-19 (31%) was significantly higher than those discharged following assessment for the same pathway during the pandemic (16%), *X*^2^ = 4.47, *p* = 0.03. There was no difference in discharge rates following assessment for the neuromodulation pathway before (4%) and during (5%) the pandemic, *X*^2^ = 0.004, *p* = 0.95. Participants who were discharged did not differ in age compared to those who were not discharged, *U* = 7339.00, *p* = 0.62. The pain duration of participants who were discharged did not differ from those who were not discharged, *U* = 3010.50, *p* = 0.25. Whether or not a patient was discharged was not significantly associated with their ethnicity (white or from an ethnically minoritized background) or gender, *X*^2^ = 0.02, *p* = 0.53 and *X*^2^ = 0.95, *p* = 0.42, respectively. Table 4 summarizes reasons for discharge and Table 5 summarizes specific details of psychosocial complexities recorded as reasons for discharge. Recommendations for onward referral/support for discharged patients are shown in Table 6.

**Table 4.** Reasons for discharge by pathway and period.

	Pre-COVID: Standard MDT pathway Frequency (%)^a^	During COVID: Standard MDT pathway Frequency (%)^b^	Pre-COVID: NM pathway Frequency (%)^c^	During COVID: NM pathway Frequency (%)^d^
**Discharge Reason:**				
Psychosocial complexities limiting engagement/safety	10 (19.6)	14 (35.9)	1 (16.7)	1 (20.0)
Not ready for self-management/seeking further pain interventions	13 (25.5)	4 (10.3)	0 (0.0)	2^e^ (40.0)
Pain not significantly impacting on functioning	6 (11.8)	4 (10.3)	1 (16.7)	0 (0.0)
Does not meet minimum physical criteria	4 (7.8)	4 (10.3)	1 (16.7)	1 (20.0)
Addiction/substance misuse	3 (5.9)	1 (2.6)	0 (0.0)	0 (0.0)
Other:				
Could not be contacted	3 (5.9)	3 (7.8)	1 (16.7)	0 (0.0)
Childcare a barrier to attending	5 (9.8)	0 (0.0)	1 (16.7)	0 (0.0)
Does not want to do group	3 (5.9)	1 (2.6)	0 (0.0)	0 (0.0)
Another PMP (condition-specific, young-adults) more suitable	0 (0.0)	4 (10.3)	0 (0.0)	0 (0.0)
Does not want to stay away from home	2 (3.9)	0 (0.0)	0 (0.0)	0 (0.0)
Unable to take time off work	1 (2.0)	0 (0.0)	0 (0.0)	0 (0.0)
Outpatient travel too much	1 (2.0)	0 (0.0)	0 (0.0)	0 (0.0)
Does not want to do virtual	0 (0.0)	1 (2.6)	0 (0.0)	0 (0.0)
Already done ACT locally	0 (0.0)	1 (2.6)	0 (0.0)	0 (0.0)
Medication side effects would interfere	0 (0.0)	1 (2.6)	0 (0.0)	0 (0.0)
Under care of another pain team	0 (0.0)	1 (2.6)	0 (0.0)	0 (0.0)
Does not want NM	0 (0.0)	0 (0.0)	1 (16.7)	0 (0.0)
Unsure if medically suitable for NM	0 (0.0)	0 (0.0)	0 (0.0)	1 (20.0)

*Note:* NM, Neuromodulation.

^a^Proportion computed from total number of discharge reasons for relevant pathway/period (^a^n = 51).

^b^n = 39.

^c^n = 6.

^d^n = 5).

^e^For NM pathway, intervention other than spinal cord stimulation.

**Table 5.** Details of psychosocial complexities recorded as reason for discharge.

	Frequency (%)^a^ collapsed across pathways and periods
Anxiety	20 (32.3)
Low mood/depression	17 (27.4)
Post-traumatic stress symptoms or trauma-related distress	13 (21.0)
Suicidality or self-harm risk	5 (8.1)
Psychosis	2 (3.2)
Living situation or social stress	2 (3.2)
Bipolar disorder	1 (1.6)
Personality disorder	1 (1.6)
Learning difficulty	1 (1.6)

*Note:* Psychosocial complexities were not mutually exclusive and more than one category could apply.

^a^Denominator for the percentage was 62, reflecting the total number of psychosocial complexities identified/coded rather than the number of patients.

**Table 6.** Recommendations made for discharged patients.

	Frequency (%)^a^ collapsed across pathways and periods
Referral to local or specialist psychological treatment	26 (39.4)
Reinforced engagement with existing/planned psychological treatment	10 (15.2)
GP review	8 (12.1)
Referral to local physiotherapy	6 (9.1)
Referral to another PMP (local, condition-specific, young-adults, etc)	6 (9.1)
Referral to local occupational therapy review	3 (4.5)
Referral to social services	3 (4.5)
Reinforced engagement with existing/planned physiotherapy	2 (3.0)
Referral to falls service	1 (1.5)
Advise at-home exercises	1 (1.5)

^a^Denominator for the percentage was 66, reflecting the total number of recommendations identified/coded rather than the number of patients.

## Discussion

The purpose of this audit was to examine assessment outcomes and reasons for exclusion from a PMP within a speciality care pain service before and during the pandemic. For the standard pain management pathway, the majority of patients were offered one of the PMP options within our service before and during the pandemic, although a greater proportion of patients was offered treatment during the pandemic. For the neuromodulation pathway, the vast majority of patients were offered one of our pre-neuromodulation PMP options, and this was similar before and during the pandemic. Psychosocial complexities and unwillingness to engage in a pain management approach that does not principally focus on pain reduction were the most common reasons that patients were not offered a programme. This audit can inform service provision and future research to optimize patient selection for virtual and in-person PMPs moving forward.

Immediately prior to the pandemic, 31% of patients were not offered a PMP on the standard pain management pathway. The discharge rates for this pathway reduced to 16% during the pandemic. This is lower than the discharge rate reported in the previous assessment outcome audit in our service conducted in 2014 (45%).^12^ In reviewing the literature, we did not identify other audits that have reported on discharge rates following assessments for PMPs from other services. Given challenges around digital poverty and literacy,^20^ a minority of patients continued to be offered an in-person PMP option during the pandemic for when treatment was allowed to be offered on site. Therefore, it appears likely that the service’s ability to offer both in-person and virtual delivery formats enabled, at least in part, a greater number of patients to engage with treatment in a manner that suited their needs. From the data available in the current audit, it is not possible to say whether virtual delivery specifically enabled people with higher levels of disability or distress to engage, or if the format was viewed as easier to engage with more generally. Nonetheless, the current findings can support the continued provision of virtual PMPs alongside in-person delivery formats to meet a wider range of needs. A key question for future research is to understand how to match patients with the delivery format that best suits their needs.

In addition to the implementation of virtual PMPs, another plausible explanation for the higher proportion of patients offered treatment in the current audit, particularly during the pandemic, was that more patients were offered a PMP for whom it was not actually suitable. However, this cannot be directly ascertained from the current audit as we were not able to link the data extracted from assessment letters with treatment engagement or outcome data. Anecdotally, clinicians observed that substantial time and effort went into organizing the virtual PMPs and keeping patients engaged with this format relative to what was typically required for the pre-pandemic residential format. Direct investigation of virtual treatment completion rates and outcomes is needed and will help elucidate whether the more inclusive assessment outcomes found in this audit actually contribute to better treatment outcomes.

It is also important to consider the role of staff turnover in shaping PMP assessment practices and outcomes. A previous study found that high periods of staff turnover predicted poorer outcomes following an intensive group-based PMP.^22^ Although the current audit did not collect data on staff turnover, the service has experienced a number of staffing changes from the 2014 audit to the current one. It may be that differences in interpreting and applying the inclusion/exclusion criteria between different staff across these time periods accounted for the more inclusive assessment outcomes observed here.

It is notable that the majority of patients on the neuromodulation pathway were offered some form of preparatory treatment within our service. The availability of a brief ‘Technology Day’ and a more intensive neuromodulation PMP thus appears to meet a range of patients’ needs. However, this audit did not further explore outcomes on the neuromodulation pathway, such as whether patients went for a trial and full implant of the stimulator. Previous research indicates that, on average, people experience improvements in functioning and mood following completion of a two-week residential neuromodulation PMP and the majority go on to receive a trial implant.^10^ Qualitative work is also needed to understand patients’ experiences at multiple points in the neuromodulation pathway, from referral to post-implantation, and to understand key barriers to and facilitators of successful outcomes from this treatment approach. Research is also needed to understand who is most likely to benefit from neuromodulation to optimize patient selection.

The current data indicate that patient discharge status was not associated with demographic factors including gender, ethnicity, or age. Interestingly, though, the standard MDT pathway consisted predominantly of women, which is consistent with the higher prevalence of many persistent pain conditions in women,^23^ while patients assessed on the neuromodulation pathway during the pre-COVID period were predominantly men. It is also notable that most patients with ethnicity data recorded were white, and this was particularly striking for patients attending assessment on the neuromodulation pathway. There was a substantial amount of missing data for ethnicity, which makes interpretation of this finding extremely challenging. Ethnicity data were extracted from the hospital record and these data are obtained centrally, not within our service. Further understanding of the reasons for which ethnicity data are not provided or recorded for a large proportion of patients within our hospital is needed. Consultation with patients, particularly those from ethnically minoritized groups,^24^ is needed to understand how to optimize collection and use of these data. When investigating potential disparities in access to pain management services, it is crucial to understand how data can be ‘weaponised against the minoritized’ (p. 2,^25^) such that the data further disadvantage or marginalize certain groups.^26^ Therefore, clear communication about the use of ethnicity data to monitor and, ultimately improve, the equity of service provision is important. It is also important to consider how demographic factors such as ethnicity might be associated with patients’ likelihood of being referred for assessment at a speciality care pain service in the first place.^26,27^

Consistent with the earlier findings by Knight et al.,^12^ the presence of complex psychosocial difficulties was one of the most common reasons for exclusion from a PMP in the current audit. Anxiety, depression and trauma-related distress that were judged by the assessing clinicians to be of a severity or complexity that would interfere with a person’s ability to safely and effectively engage in a PMP were the most common psychosocial complexities indicated as a reason for programme exclusion. This is mirrored in randomized-controlled trials of psychological treatments for pain, where people with severe mental health problems are often excluded despite the comorbidity of these problems with chronic pain.^28,29^ There is a growing body of research showing the benefits of simultaneously treating post-traumatic stress and chronic pain in particular.^30-35^ Given synergies in cognitive-behavioural treatments for pain and mental health, research is needed to better understand how best to integrate treatments for both of these difficulties to further promote inclusion.^29^

In the current audit, onward recommendations were frequently made for patients who were not offered treatment in our service, including recommendations about engaging in local (trauma-focused) psychological therapy. However, there are barriers to patients accessing these treatments, such as whether an appropriate mental health service is available locally and clinicians’ knowledge of and capacity to refer to such services. Therefore, further work is needed to understand the extent to which such recommendations are actioned and, most importantly, benefit patients. Further collaboration between pain management and mental health services is needed to optimize care for this group. This might include, for example, the provision of pain management training within mental health services or developing more direct referral pathways between services. Effectiveness and implementation research are needed^29^ to understand whether trauma-focused treatment for people with pain is optimally delivered within pain management services, or in mental health services with training in pain-informed trauma therapy. In addition to providing more holistic care for patients, such a combined approach has the potential to reduce referrals between currently siloed services and, ultimately, may reduce costs, although this requires testing.

Consistent with the findings of Knight et al.,^12^ a common reason for discharge was that patients were not yet willing to engage in a pain management approach as they wished to focus on pursuing other investigations or treatments to control the pain. In reviewing the assessment letters, recommendations for discharged patients often indicated the possibility of re-referral to our service in the future. Although challenging, it would be informative to audit the longer-term referral and assessment outcomes of these patients to see the proportion who return to the service and engage in a PMP at a later date. To increase service efficiency and improve patients’ experience, there remains a need to optimize the referral process to ensure that patients are not referred for a PMP assessment at a time when they are unlikely to be able to engage with and benefit from this approach. Involvement of patients in the design of pre-referral information resources could ensure that they can make a more informed decision about whether and when referral for an assessment for a PMP would be most helpful for them. Research is also needed to understand how to best holistically support patients while they are still seeking medical solutions for pain management.

Several limitations must be considered. As mentioned, there was a substantial amount of missing data on ethnicity. Therefore, it is not possible to determine whether assessment outcomes were associated with ethnicity with certainty. The data represent a snapshot in time immediately before and in the early months of the pandemic. With increasing experience assessing for and delivering virtual programmes, it is plausible that current assessment outcomes and reasons for exclusion changed over the later months of the pandemic. The reasons for exclusion were obtained from the assessment letters. However, a complexity of information is considered when making decisions about treatment that may not be completely documented in the assessment letters. Standardized self-report questionnaire data on pain-related disability and distress were not collected as part of the multidisciplinary assessment. Because of limitations in the data available within the assessment letters, we are not able to speak directly to the important issue of whether patient presentation changed during the pandemic or whether there was a change in clinicians’ willingness to offer a PMP with the availability of a virtual format. Standardized pain and psychosocial questionnaires are collected for participants who go on to complete PMPs within the service and a separate evaluation of participant characteristics based on these data is ongoing. Finally, this audit is also unable to speak to factors that influence whether patients are referred for assessment in the first place.

Despite these limitations, the current data point to a pattern of more inclusive assessment outcomes at a specialty pain service over time and particularly during the COVID-19 pandemic. Offering a range of in-person and virtual pain management programmes is likely to enable services to be more inclusive and meet a wider range of patient need. Research is needed to understand how to best assess and match patients with the breadth of treatment delivery formats now available. Outcome data from virtual programmes also need to be scrutinized. Greater understanding of factors that exclude patients from even being referred for assessment is needed. Further research to optimize treatment pathways and outcomes for people with complex mental health problems is needed.
